# Age by Single Nucleotide Polymorphism Interactions on Bronchodilator Response in Asthmatics

**DOI:** 10.3390/jpm11010059

**Published:** 2021-01-19

**Authors:** Kirsten Voorhies, Joanne E. Sordillo, Michael McGeachie, Elizabeth Ampleford, Alberta L. Wang, Jessica Lasky-Su, Kelan Tantisira, Amber Dahlin, Rachel S. Kelly, Victor E. Ortega, Sharon M. Lutz, Ann C. Wu

**Affiliations:** 1 Department of Population Medicine, Harvard Pilgrim Health Care Institute and Harvard Medical School, Boston, MA 02215, USA; Kirsten_Voorhies@harvardpilgrim.org (K.V.); rejoa@channing.harvard.edu (J.E.S.); smlutz@hsph.harvard.edu (S.M.L.); 2 Channing Division of Network Medicine, Brigham and Women’s Hospital, Boston, MA 02115, USA; remmg@channing.harvard.edu (M.M.); reawa@channing.harvard.edu (A.L.W.); jessica.a.su@gmail.com (J.L.-S.); rekgt@channing.harvard.edu (K.T.); readh@channing.harvard.edu (A.D.); hprke@channing.harvard.edu (R.S.K.); 3 Department of Internal Medicine, Wake Forest School of Medicine, Winston-Salem, NC 27101, USA; eamplefo@wakehealth.edu (E.A.); vortega@wakehealth.edu (V.E.O.); 4 Division of Pediatric Respiratory Medicine, Department of Pediatrics, University of California San Diego, San Diego, CA 92093, USA; 5 Department of Biostatistics, Harvard T.H. Chan School of Public Health, Boston, MA 02115, USA; 6 Division of General Pediatrics, Department of Pediatrics, Children’s Hospital, Boston, MA 02215, USA

**Keywords:** bronchodilator response, genome-wide interaction study, asthma

## Abstract

An unaddressed and important issue is the role age plays in modulating response to short acting β2-agonists in individuals with asthma. The objective of this study was to identify whether age modifies genetic associations of single nucleotide polymorphisms (SNPs) with bronchodilator response (BDR) to β2-agonists. Using three cohorts with a total of 892 subjects, we ran a genome wide interaction study (GWIS) for each cohort to examine SNP by age interactions with BDR. A fixed effect meta-analysis was used to combine the results. In order to determine if previously identified BDR SNPs had an age interaction, we also examined 16 polymorphisms in candidate genes from two published genome wide association studies (GWAS) of BDR. There were no significant SNP by age interactions on BDR using the genome wide significance level of 5 × 10^−8^. Using a suggestive significance level of 5 × 10^−6^, three interactions, including one for a SNP within *PRAG1* (rs4840337), were significant and replicated at the significance level of 0.05. Considering candidate genes from two previous GWAS of BDR, three SNPs (rs10476900 (near *ADRB2*) [*p*-value = 0.009], rs10827492 (*CREM*) [*p*-value = 0.02], and rs72646209 (*NCOA3*) [*p*-value = 0.02]) had a marginally significant interaction with age on BDR (*p* < 0.05). Our results suggest age may be an important modifier of genetic associations for BDR in asthma.

## 1. Introduction

Asthma affects more than 330 million people around the world [1]. The projected economic burden of uncontrolled asthma in the US alone is estimated to be 300 billion dollars over the next 20 years [2]. Asthma is often treated with β2-agonists (e.g., albuterol), to relieve acute bronchoconstriction [3]. β2-Agonists reduce bronchoconstriction by stimulating the β2-adrenergic receptors (β2ARs) on airway smooth muscle. Bronchodilator response (BDR) measures the change in airway constriction before and after the administration of a short-acting β2-agonist (SABA). High inter-individual variability exists in the response to inhaled β2-agonists due to genetics [4], with heritability of BDR ranging from 10–40% [5,6,7].

Genetic associations differ for childhood versus adult-onset asthma [8], yet most studies in asthma pharmacogenetics ignore the potential for age-related heterogeneity, combining data from children, adolescents, and adults. Known associations between genes and asthma drug response suggest that specific genetic mechanisms regulating BDR may vary with age. For example, variants in *ARG1*, a gene known to modulate BDR, may have age related effects [9].

Age may be an important modifier of pharmacogenetic associations especially because age-related differences in asthma phenotypes may reflect different underlying pathogenic pathways [10]. Given that few studies have examined age by SNP interactions on BDR, we examined these interactions in the Childhood Asthma Management Program (CAMP) [11], the Childhood Asthma Research and Education trial (CARE) [12,13], and the Effectiveness of Low Dose Theophylline as an Add-on Treatment in Asthma trial (LODO) studies [14]. We also considered two previously published GWAS of BDR in order to examine if SNPs associated with BDR also interacted with age [9,15].

## 2. Materials and Methods

### 2.1. Study Populations

The discovery population was composed of three independent cohorts: CAMP, CARE and LODO (total *n* = 892). CAMP was a pediatric asthma trial with subjects age 5 to 12 years at enrollment with “mild to moderate asthma”; 1041 subjects were enrolled and received treatment for 4 to 6 years [11]. Subjects were randomly assigned to receive budesonide, nedocromil, or placebo. We included two of the five CARE trials, the Pediatric Asthma Controller Trial (PACT) [12] and Characterizing Response to Leukotriene Receptor Antagonist and Inhaled Corticosteroid (CLIC) [13]. LODO was a clinical trial of adults with poorly controlled asthma who were randomly assigned to placebo, theophylline, or montelukast [14]. Only subjects of European ancestry were included.

We conducted a meta-analysis including 892 subjects from these 3 cohorts: 560 subjects from CAMP, 206 subjects from CARE, and 126 subjects from LODO. All study procedures were approved by the respective Institutional Review Boards of each consortium and the Brigham and Women’s Hospital.

For replication, we used data from 559 subjects in the Severe Asthma Research Program (SARP). SARP was a study of pediatric and adult subjects with mild, moderate, or severe asthma [16]. “Maximal” lung function included responsiveness between two to eight puffs of albuterol (SARP1-2 started at two puffs and SARP3 started at four puffs then both increased until maximum bronchodilation was attained). 

### 2.2. Outcomes

The main outcome for this study was BDR using baseline measurements for each cohort. BDR was measured similarly in all cohorts. Calculation of BDR was based on pre- and post-bronchodilator forced expiratory volume at one second (FEV_1_) measurements. After two puffs of albuterol by Metered Dose Inhaler (MDI) with spacer was administered, at least 10 min elapsed before the post-bronchodilator spirometry was performed. β2-agonist response (bronchodilator response (BDR)) = 100 × [(post FEV_1_ − pre FEV_1_)/pre FEV_1_]. 

### 2.3. Genotyping, Imputation and Quality Control Procedures

Genotyping procedures in CAMP, CARE and LODO have been previously described [17]. Standard QC procedures were applied to the merged, imputed dataset to remove markers with low minor allele frequency (<5%). Principal components analysis (PCA) was performed using PLINK. A final dataset of 5,638,778 typed and imputed markers and 892 subjects passed all sample and genotype QC measures for analysis.

### 2.4. Statistical Analyses

We ran a genome wide interaction (GWI) analysis for each of the three cohorts in PLINK [18], examining the interaction of age and SNPs on BDR, adjusting for age, sex, BMI as a categorical variable (obese, overweight, vs. normal/underweight), and genetic ancestry via principal components (PCs). For genetic ancestry, we adjusted for 3 PCs in the CAMP and LODO cohorts and 5 PCs in the CARE cohort. We determined the number of principal components to adjust for our statistical models based on scree plots and the proportion of variance explained. We then meta-analyzed the age by SNP interactions on BDR for the three cohorts in METAL weighting by sample size [19].

## 3. Results

**Figure 1:**
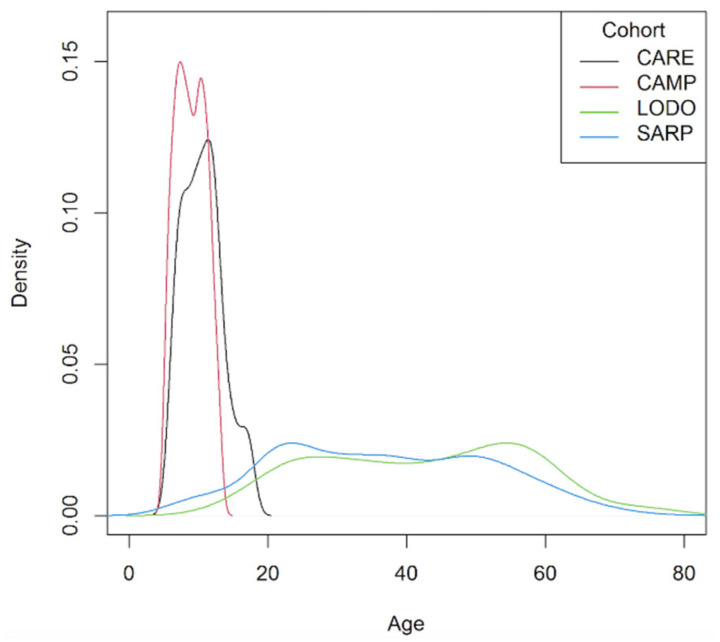
Distribution of ages for subjects from CARE, CAMP, LODO, and SARP.

Table 1 shows characteristics of participants from each cohort. Overall, participants ranged in age from 5.2 to 78.2 years. The distribution of ages for the four cohorts are shown in Figure 1. The CAMP and CARE studies were composed of childhood participants with asthma (mean ± SD of ages were 8.9 ± 2.1 and 10.6 ± 2.9 years, respectively), while the LODO and SARP studies mainly consisted of adults with asthma (42.4 ± 15.1 and 36.9 ± 15.3, respectively). Average BDR levels were similar across CAMP, CARE, LODO, and SARP. 

In our GWI analyses, we did not find any significant SNP by age interactions on BDR when considering a genome wide significance level of 5 × 10^−8^. Using a suggestive significance level of 5 × 10^−6^, we found 30 significant SNP by age interactions on BDR. Three of these SNPs replicated in the SARP cohort at a significance level of 0.05. Replications were directionally consistent with different minor alleles in the meta-analysis cohorts and replication cohort. Results are shown in Table 2. Two of these SNPs are in gene deserts and 1 SNP is located on chromosome 8 in gene *PRAG1*.

Using 16 candidate SNPs from two published GWAS of BDR [9,15] we examined if these SNPs had an interaction with age on BDR. We picked these two manuscripts for candidate regions since the first manuscript [9] found 5 regions associated with BDR in similar cohorts of 962 children (CAMP, the Asthma trial [9], the Leukotriene Modifier or Corticosteroid or Corticosteroid Salmeterol trial (LOCCS), and LODO) and the second manuscript [15] found six novel regions and examined six candidate regions associated with BDR among 1782 Latino children. Three of these candidate SNPs had a significant interaction with age on BDR (Table 3). These three SNPs were located on chromosomes 5 [*ADRB2*], 10 [*CREM*], and 20 [*NCOA3*].

**Figure 2:**
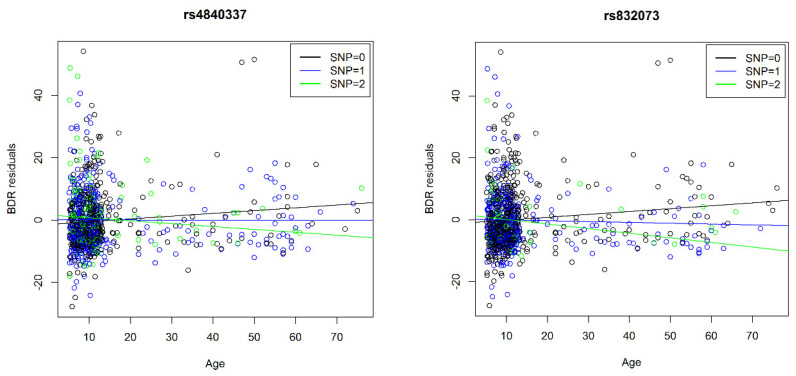

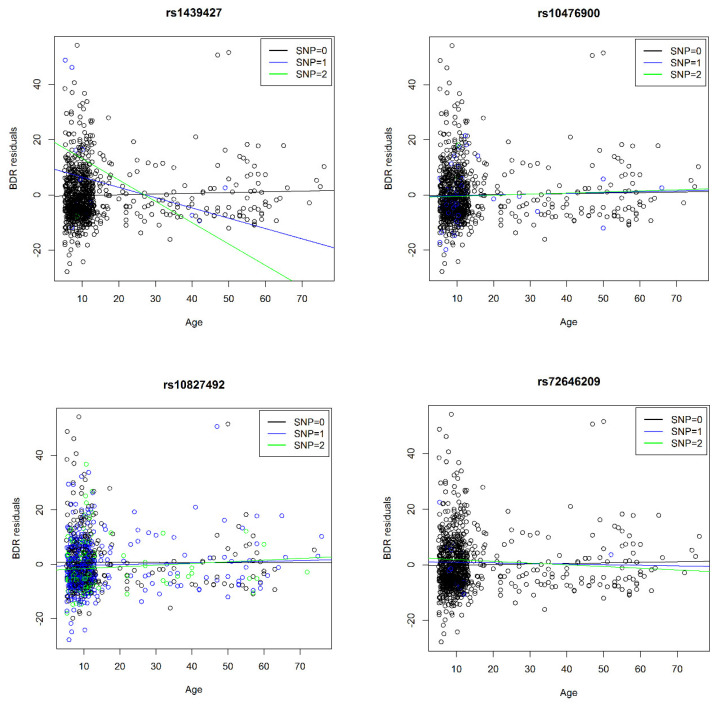
The plots below show the effect of the six SNPs on the association between age and BDR regressing age, sex, obese, overweight, cohort and genetic ancestry. Note that the minor allele frequency for rs1439427, rs10476900, rs72646209 is less than 0.05.

There was a positive but non-significant correlation between age and BDR: 0.04 (*p* = 0.33) in CAMP, 0.04 (*p* = 0.59) in CARE, and 0.1 (*p* = 0.28) in LODO. In Figure 2, we examined the top three SNPs from the main analysis (rs832073, rs4840337, rs1439427 [Table 2]) and the top three candidate SNPs (rs10476900, rs10827492, rs72646209 [Table 3]). Figure 2 shows the effect of the six SNPs on the association between age and BDR regressing cohort, age, sex, BMI as a categorical variable (obese, overweight, vs. normal/underweight), and genetic ancestry. Note there was a low minor allele frequency for rs1439427, rs10476900, rs72646209. Given the small sample sizes, these results should be interpreted with caution. Studies with larger sample sizes are needed to further examine these SNP by age interactions on BDR.

## 4. Discussion

Our study on age by genotype interactions for BDR in asthma has two key findings. First, we uncovered three novel SNPs (one in *PRAG1*) that interacted with age at a suggestive genome wide significance level of 5 × 10^−6^, with replication (*p* < 0.05) of our findings in an external cohort. Second, we then tested SNPs from prior GWAS studies for interaction with age in our cohort and three variants in the genes *CREM*, *NCOA3* and near *ADRB2* showed differential associations at a marginal significance level of 0.05 with BDR by age. These results suggest that accounting for age-related modification of genetic associations may aid in the detection of novel pharmacogenetic variants, and may refine our understanding of known genetic variants for the BDR phenotype given the context of age. 

While there were no significant age by SNP interactions on BDR at the genome wide significance level of 5 × 10^−8^, there were three SNPs that were suggestive signals at the 5 × 10^−6^ level that replicated in the SARP cohort at a significance level of 0.05. Two of these SNPs are in gene deserts. One SNP was in gene *PRAG1* on chromosome 8. *PRAG1* has previously been linked to psychological outcomes, including selective serotonin reuptake inhibitors remission and neuroticism [20], however this is the first report to identify an association of *PRAG1* with an asthma-related phenotype. 

In order to examine whether SNPs previously associated with BDR had a significant age interaction on BDR, we considered two GWAS of BDR. We found three significant age by SNP interactions with BDR. These three SNPs were located on chromosomes 5 [near *ADRB2*], 10 [*CREM*], and 20 [*NCOA3*]. *ADRB2* [21] has previously been associated with asthma and *NCOA3* is involved in transcriptional regulation and has previously been associated with BDR in Puerto Ricans with asthma [15]. While *ADRB2* (the gene that encodes the direct target of SABAs) is one of the most widely studied candidate genes for BDR, its associations with this phenotype have been inconsistent [22], perhaps because studies thus far have not accounted for interactions between *ADRB2* variants and age. *CREM* has been associated with Th-2 mediated inflammation in asthma [23] and, given the findings in this study, may potentially play a role in age-specific asthma treatment outcomes.

Strengths of our study include the use of data from multiple clinical trials with a wide span of ages ranging from childhood to adulthood. To our knowledge, this is the first meta-analysis to identify potential age-by-genotype interactions for bronchodilator response in asthma. Despite the strengths of our study, a few limitations deserve mention. Even though the sample size is large for a pharmacogenomics GWAS, larger samples sizes would have enhanced the statistical power to detect interactions. Moreover, our study was limited to subjects of European ancestry which limits the generalizability of our findings, although this limitation helped prevent issues with population stratification. Also, there was a low minor allele frequency for rs1439427, rs10476900, rs72646209; given the small sample size, these results should be interpreted with caution. Studies with larger sample sizes are needed to further examine these SNP by age interactions on BDR.

In conclusion, it is important to consider age in pharmacogenetic studies and age should be examined both in GWI analyses and as a potential effect modifier of genes within known asthma treatment response pathways. A clear understanding of how age impacts the association of the genotype with BDR has implications for the pharmacogenomics of asthma treatment responses. Understanding how age modifies genetic effects can help tailor pharmacogenomic testing in an age-specific manner, which will ultimately have clinical importance in personalized medicine. For example, albuterol as a rescue medication may have worked well during childhood for an individual with asthma, but may not be as effective during adulthood. Thus, providers may need to consider other rescue or controller medications at different ages. Developing clinical recommendations is beyond the scope of this analysis; however, this study demonstrates that accounting for age-related modification of genetic associations needs further evaluation.

## Figures and Tables

**Table 1:** Characteristics of subjects from CARE, CAMP, LODO, and SARP.

	Meta-Analysis Cohorts			Replication Cohort
	CARE (*n* = 206)	CAMP (*n* = 560)	LODO (*n* = 126)	SARP (*n* = 559)
Age mean years (SD)	10.6 (2.9)	8.9 (2.1)	42.4 (15.1)	36.9 (15.3)
Age range	6.0–17.8	5.2–13.2	15.0–76.0	6.4–78.2
Sex (female), *n* (%)	78 (37.9)	225 (40.2)	94 (74.6)	366 (65.5)
Overweight, *n* (%)	37 (18.0)	87 (15.5)	32 (25.4)	160 (28.6)
Obese, *n* (%)	38 (18.4)	78 (13.9)	47 (37.3)	191(34.2)
BDR, mean (SD)	9.7 (8.4)	10.9 (10.3)	8.7 (10.2)	11.3 (13.4)

**Table 2:** Significant results from the meta-analysis of CAMP, CARE, and LODO that replicated in SARP.

Meta-Analysis (CAMP, CARE, LODO)																SARP			
Chr	Gene	Position/rs#	*n*	MAF CAMP	MAF CARE	MAF LODO	Beta CAMP	SE CAMP	Beta CARE	SE CARE	Beta LODO	SE LODO	A1	Z-Score	*p*	Position/rs#	A1	Beta	*p*
3		97697002/rs832073	892	25.3%	23.3%	27.8%	−1.3	0.3	−0.7	0.3	−0.1	0.1	T	4.6	3 × 10^−6^	97697002/rs832073	G	−0.1	0.02
8	*PRAG1*	8198306/rs4840337	892	33.8%	33.7%	38.5%	−1.3	0.3	−0.4	0.3	−0.1	0.1	C	4.8	1 × 10^−6^	8198225/rs2945913	T	−0.2	0.04
18		1845637/rs1439427	560	0.8%	0.7%	1.2%	−7.9	1.7	NA	NA	NA	NA	A	−4.6	3 × 10^−6^	1846172/rs8091804	C	−0.2	0.006

**Table 3:** Results for candidate SNPs.

Meta-Analysis (CAMP, CARE, LODO)																Candidate GENES					
Chr	Gene/Nearest Gene	Position/rs#	*n*	MAF CAMP	MAF CARE	MAF LODO	Beta CAMP	SE CAMP	Beta CARE	SE CARE	Beta LODO	SE LODO	A1	Z-Score	*p*	Position/rs#	A1	MAF	Beta	*p*	
1	*PAPPA2*	176695479/rs77977790	892	5.9%	6.8%	6.0%	−0.3	0.6	0.7	0.6	0.1	0.2	T	−0.4	0.7	176695479/rs77977790	-	2.8%	9.5	5 × 10^−10^	*
2	*SPATS2L/KCTD18*	201354935/rs3795969	892	41.6%	37.8%	36.1%	0.2	0.3	−0.7	0.3	0.1	0.1	C	0.2	0.9	201354866/rs10203042	-	2.1%	4.1	2 × 10^−3^	*
3	*THRB*	24573150/rs73038406	766	2.4%	3.2%	0.8%	−1.7	1.0	0.6	0.7	NA	NA	T	−1.0	0.3	24573150/rs73038406	-	1.5%	−4.0	0.01	*
5	*ADRB2*	148162955/rs10476900	892	3.0%	2.9%	2.4%	1.6	1.0	2.7	1.1	0.1	0.3	A	−2.6	9 × 10^−3^	148162955/rs10476900	-	10.3%	1.3	0.04	*
6	*intergenic*	28619532/rs116551936	892	1.6%	1.5%	1.2%	−0.9	1.3	1.6	1.1	0.1	0.4	A	0.3	0.8	28619532/rs116551936	-	0.3%	27.0	6 × 10^−9^	*
6	*ARG1*	131891820/rs2781659	892	32.5%	31.3%	31.3%	−0.1	0.3	0.1	0.3	−0.1	0.1	A	0.4	0.7	131891820/rs2781659	A	-	-	5 × 10^−4^	♦
6	*IGF2R*	160429357/rs8191725	892	2.9%	2.9%	3.2%	−0.5	1.0	1.1	1.0	−0.1	0.3	A	0.0	1.0	160429357/rs8191725	-	0.8%	10.2	3 × 10^−9^	*
7	*CRHR2*	30719049/rs1003929	892	15.9%	14.3%	12.7%	0.7	0.4	−0.3	0.4	0.1	0.1	T	1.2	0.2	30719049/rs1003929	C	-	-	0.01	♦
10	*CREM*	35429825/rs10827492	892	36.3%	33.5%	36.1%	0.9	0.3	0.0	0.3	0.0	0.1	T	2.3	2 × 10^−2^	35429825/rs10827492	C	-	-	0.05	♦
11	*SPON1*	14085131/rs77149876	686	0.6%	0.2%	1.2%	−0.2	2.2	NA	NA	0.4	0.4	T	−0.3	0.7	14085131/rs77149876	-	0.2%	32.5	1 × 10^−8^	*
11	*intergenic*	96962052/rs74973995	892	1.6%	1.7%	0.8%	−0.4	1.1	0.9	1.4	0.0	0.4	A	0.0	1.0	96962052/rs74973995	-	0.2%	32.6	1 × 10^−8^	*
12	*CREBL2*	12796872/rs4555	892	55.8%	54.9%	57.1%	−0.4	0.3	0.3	0.3	−0.1	0.1	T	0.9	0.4	12796872/rs4555	A	-	-	0.05	♦
12	*CPM*	69309377/rs1144961	892	25.4%	24.8%	25.8%	−0.3	0.4	−0.3	0.4	0.1	0.1	A	−0.7	0.5	69309377/rs1144961	G	-	-	0.05	♦
14	*SLC24A4*	92960148/rs4900131	892	14.9%	13.1%	17.9%	−0.6	0.4	−0.4	0.5	0.0	0.1	T	−1.7	0.1	92959857/rs77441273	-	0.2%	23.6	4 × 10^−10^	*
16	*ADCY9*	4192082/rs7201216	892	4.6%	3.2%	3.2%	0.5	0.7	0.9	1.0	−0.1	0.3	A	−0.9	0.4	4191522/rs144315541	-	0.7%	−6.7	4 × 10^−4^	*
20	*NCOA3*	46282645/rs72646209	766	0.5%	1.7%	0.4%	−3.9	2.1	−2.9	1.9	NA	NA	A	−2.4	2 × 10^−2^	46282708/rs115501901	-	0.2%	31.5	4 × 10^−8^	*

Note that the allele, MAF, and Beta were not available in all candidate published papers. * SNP from Drake et al. 2014 [15] and ♦ SNP from Litonjua et al. 2008 [9]

## Data Availability

The data presented in this study (CAMP, CARE, and LODO cohorts) are openly available in dbGaP at https://www.ncbi.nlm.nih.gov/projects/gap/cgi-bin/study.cgi?study_id=phs000166.v2.p1.
